# Magnetic Resonance Imaging of Alimentary Tract Development in *Manduca sexta*

**DOI:** 10.1371/journal.pone.0157124

**Published:** 2016-06-09

**Authors:** Ian J. Rowland, Walter G. Goodman

**Affiliations:** Department of Entomology, University of Wisconsin-Madison, Madison, Wisconsin, United States of America; Natural Resources Canada, CANADA

## Abstract

Non-invasive 3D magnetic resonance imaging techniques were used to investigate metamorphosis of the alimentary tract of *Manduca sexta* from the larval to the adult stage. The larval midgut contracts in volume immediately following cessation of feeding and then greatly enlarges during the late pharate pupal period. Magnetic resonance imaging revealed that the foregut and hindgut of the pharate pupa undergo ecdysis considerably earlier than the external exoskeleton. Expansion of air sacs in the early pupa and development of flight muscles several days later appear to orient the midgut into its adult position in the abdomen. The crop, an adult auxiliary storage organ, begins development as a dorsal outgrowth of the foregut. This coincides with a reported increase in pupal ecdysteroid titers. An outgrowth of the hindgut, the rectal sac, appears several days later and continues to expand until it nearly fills the dorsal half of the abdominal cavity. This development correlates with a second rise in pupal ecdysteroid titers. In the pharate pupa, the presence of paramagnetic species renders the silk glands hyperintense.

## Introduction

The insect alimentary tract is a dynamic and complex structure responsible for processing and sequestering molecules of nutritional value, maintaining and/or excreting metabolites [1], harboring symbionts that aid nutrition [2], secreting defensive compounds [3], preventing microbial infections [4], [5], limiting xenobiotic damage [6], and secreting hormones [7, 8].

The alimentary tract of Lepidoptera is a remarkable organ not only for its relative size in relation to the body during the larval stage but its transformation during metamorphosis to the pupal and adult stages. Developmentally, the alimentary tract of Lepidopterans undergoes significant restructuring during metamorphosis as the insect transitions from feeding on foliage to nectar [9–11]. While there is a fairly complete picture of the cellular and ultrastructural events surrounding the restructuring of the tract, temporal appearance of macroanatomical structures such as the crop or rectal sac have been largely ignored. Moreover, gross dissection of the alimentary tract may suffer from physical displacement of tissues and organs induced by invasive manipulation. These distortions can lead to misrepresentation of the actual location or anatomical context within the insect. While studies using X-ray imaging based methods have added to our understanding of insect anatomy, MRI offers significant advantages due to its inherent soft tissue contrast [12–31].

The present study employs MRI to examine the metamorphosis of the alimentary tract, silk glands and mandibular glands in the Lepidopteran *Manduca sexta* (tobacco hornworm). Throughout the study, high-resolution imaging using parameters sensitive to the presence of paramagnetic species has been utilized to identify tissues that appear hyperintense due to the natural presence of paramagnetic species. Identification of such species could lead to the development of novel, positive-contrast non-mammalian MRI reporter genes.

This MRI study provides a unique and new perspective on developmental changes in the alimentary tract of a single insect, *Manduca sexta*, following the tract’s significant restructuring. Unlike the foregut and hindgut, which are shed during ecdysis and pupation, the midgut is retained and remains a target for pesticides throughout the insect’s lifetime. Moreover, the employment of MR imaging reveals, for the first time, the dynamic nature of the morphological changes occurring during the larval, pupal and adult stages while relating those changes with endocrinological events in a single species.

## Materials and Methods

### Insect rearing and MRI preparation

*Manduca sexta* (Madison strain, [32]) were reared under a 16:8 L:D photoregime at 26°C. Developmental staging of larvae and pupae was performed using the chronological age of the insect coupled with morphological markers described by Goodman et al. (32). Insects were fed on commercially available gypsy moth wheat germ diet (MP Biomedicals). To aid MR visualization of the alimentary tract, some animals received a diet containing contrast agent (CA; Omniscan, GE Healthcare, final concentration 5 mM gadodiamide). Some larvae were fed diets containing high levels of CA (final concentration 50 mM) for 2 days prior to entering the post-feeding stage. This ensured that a significant amount of CA remained in the alimentary tract even after gut purging. Adults were fed sugar water (20% sucrose) containing CA (0.5 mM). The sugar water was supplemented with red food coloring (McCormick) to confirm that the insect had fed.

### MRI

All larvae, pupae and adult insects were immobilized by placing them in a plastic syringe barrel, immersing them in perflurocarbon (Fluorinert, 3M^™^), and removing surrounding trapped air using a slight vacuum. Perfluorcarbon immersion has the advantage of a much-reduced magnetic susceptibility difference when compared to imaging the insect in air. Insects were then centrally positioned in a Varian quadrature volume coil (3 cm inner diameter) and images acquired at 4.7T using a Varian Inova horizontal imaging and spectroscopy system (Agilent). The self-shielded scanner utilized a gradient system with an inner bore of 12cm and a maximum strength of 100 mT/m. All MR data were acquired within an 8-hour imaging time period. A three-dimensional gradient echo (3D-GRE) sequence (TR = 20.6 ms; TE = 6.5 ms; FL = 65°; FOV = 60 x 15 x 15 mm or 30 x 15 x 15 mm; MA = 512 x 256 x 256; NT = 16) was used to acquire 3D data. Spin echo 2D (2D-SE) slices were also acquired (TR = 2100 ms; TE = 17 ms; FOV = 15 x 15 mm; MA = 256 x 256; SL = 1 mm; NT = 8). For some animals, 3D images were acquired using a fast spin echo (3D-FSE) sequence (TR = 220 ms; TE_eff_ = 60 ms; FOV = 60 x 15 x 15 mm; MA = 512 x 256 x 256; NT = 16). ImageJ [33] was used to convert acquired data into 8-bit Analyze format and to construct two-dimensional images, re-slice the data and obtain linear measurements. Three-dimensional surface renderings were obtained using Osirix software [34] following importation of data in Analyze format. Different insects were used in each image unless otherwise noted. Photoshop (Adobe, V CS5.1), was used to prepare the figures. Insect outlines were prepared using Photoshop and photographs of larva, pupae and adults. Where ambiguities existed in the interpretation of images, additional insects were studied further. Dissections and visual examinations were performed to confirm the MRI observations. To obtain the relative sizes and positions of the organs, a combination of imaging and dissection findings were used. The 3D imaging data was re-sliced, using ImageJ, to obtain a longitudinal slice through the middle of the insect. The image was scaled to match the outline of the insect and organs were ‘traced’ using Photoshop. Where organs eg proboscis could not be readily observed using MRI, dissection was used to obtain exact locations.

## Results

### Larval feeding period

**Fig 1:**
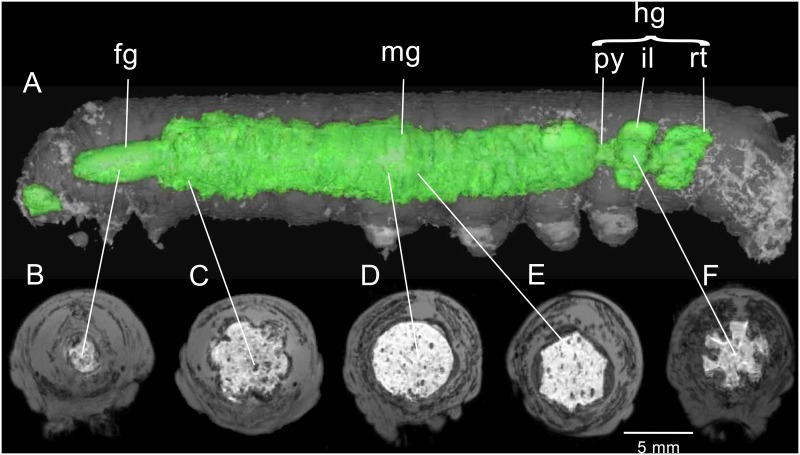
3D-GRE MR imaging data acquired from a 92 hour feeding fifth instar *M*. *sexta*. (A) Longitudinal surface-rendered view showing the digestive tract, including the foregut (fg), midgut (mg) and hindgut (hg), the latter consisting of the pylorus (py), ilium (il) and rectum (rt). Lack of CA between the buccal cavity and the esophagus may be due to swallowing. The insect has just defecated. White lines connect regions in the longitudinal view with corresponding transverse sections. (B) Foregut region. (C) Anterior midgut region, with prominent out-pocketings. (D) Middle midgut, displaying rounded lumen. (E) Middle midgut, displaying hexagonal-shaped lumen. (F) Hindgut, displaying the ileal nodes.

Fig 1 is a composite of MR images acquired from a feeding fifth instar (92 h post-ecdysis) fed diet containing contrast agent (CA). Fig 1A displays a lateral view revealing the intraluminal cavities of the foregut, midgut and hindgut. The foregut extends from the head to the 3^rd^ thoracic segment while the midgut extends to the third pair of prolegs (6^th^ abdominal segment). The alimentary tract at the midgut-hindgut junction tapers to form the pylorus and it is here that Malpighian tubules enter the tract (not shown). Posterior to the pylorus is the ileum, where fecal pellets are formed [35]. The ilium is characterized by its lobed nature. Posterior to the ilium is the rectum (7-10^th^ abdominal segments).

Transverse sections at various levels of the alimentary tract reveal differences in size and shape. The foregut has a smooth luminal surface (Fig 1A and 1B) and is surrounded by a thin dark line which we interpret as muscle (Fig 1B). The anterior portion of the midgut displays 6 out-pocketings not observed in the rest of the midgut (Fig 1C); it appears that longitudinal muscles terminate on the foregut, inducing formation of these structures. The luminal surface of the anterior midgut appears more rugose than the rest of the midgut and in transverse sections appears to be invested in a layer of circular muscles (Fig 1C).

Serial transverse sections of the middle portion of the midgut reveal two seemingly different anatomical configurations, a circular configuration (Fig 1D) and a hexagonal configuration (Fig 1E). The image in Fig 1D illustrates a transverse section of the midgut in the region between the bands of circular muscles where the wall expands outward; the outward folds create the corrugated appearance of the midgut when viewed from outside. Fig 1E represents the region where circular muscles constrict the diameter of the midgut (Fig 1A). The thickened walls of the hexagonal-shaped midgut represent both the circular muscles and midgut cells that have been compressed inward by the overlying muscles. Six longitudinal muscles can be seen at the angles formed by the hexagon. Numerous large gas bubbles (dark regions) appear embedded in the midgut contents (Fig 1C–1E). These inclusions were rarely seen in the foregut or in insects that had cleared their gut contents in preparation for metamorphosis.

The highly structured hindgut, which has a smaller diameter than the midgut, is illustrated in Fig 1F. Six ridges from the hindgut wall project into the food stream and are oriented parallel with the tract. A partially formed frass pellet can be seen in the ileum (Fig 1A). The tract ends in an enlarged rectum and in Fig 1A it has been recently voided.

### Post-feeding larval period

**Fig 2:**
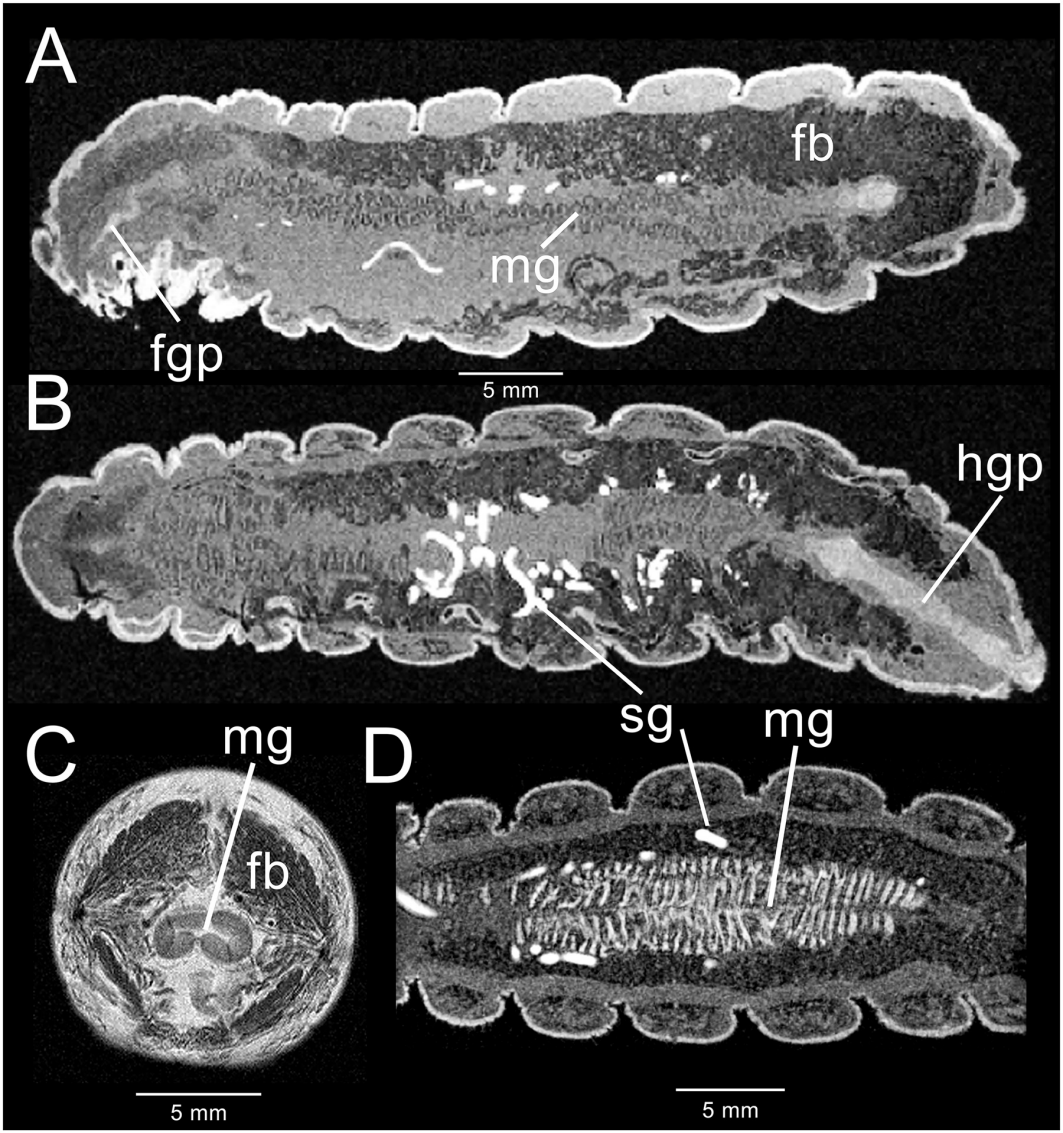
MR imaging data acquired from two different 180 hour fifth instar *M*. *sexta*. The insect shown in A-C was not previously fed CA; the insect shown in D was previously fed CA. (A) 3D-GRE mid-sagittal section displaying the fat body (fb), foregut “plug” (fgp) and highly shrunken midgut. (B) Coronal section of the same insect, revealing the silk glands (sg) and hindgut “plug” (hgp). (C) Transverse 2D-SE slice of the same insect through the second abdominal segment, showing collapse of the midgut. (D) 3D-GRE coronal section through abdominal segments 3–6, displaying the collapsed midgut and the silk glands.

After the fifth instar purges its gut contents in preparation for metamorphosis, the alimentary tract shrinks in relation to body mass (compare Fig 1 with Fig 2) and becomes flattened on a dorso-ventral axis (Fig 2C). As it flattens, the midgut appears to push into the thoracic region as far as the mesothoracic segment. The circular muscles that gave rise to the corrugated surface of the midgut in the feeding larva now appear reduced in number (Fig 2B and 2D). Ventral regions once occupied by the midgut become filled with an unknown mass, while regions surrounding the hindgut become more heavily invested with fat body (Fig 2A and 2C). During the process of shrinkage, the foregut lumen becomes folded, exhibiting a MR signal intensity isointense with the cuticle. This region has the appearance of a plug (Fig 2A). A portion of the plug-like structure in the foregut appears to push into the midgut. At the same time, a plug-like structure appears in the hindgut. Gross dissection of the foregut and hindgut lumens reveals a free-floating, lightly tanned membranous structure that may represent shed cuticle. This tanned, free-floating cuticle may account for the isointensity of the foregut and hindgut with the exoskeleton. The rectum is considerably reduced in volume, leaving only the ileum to show remnants of larval character.

**Fig 3:**
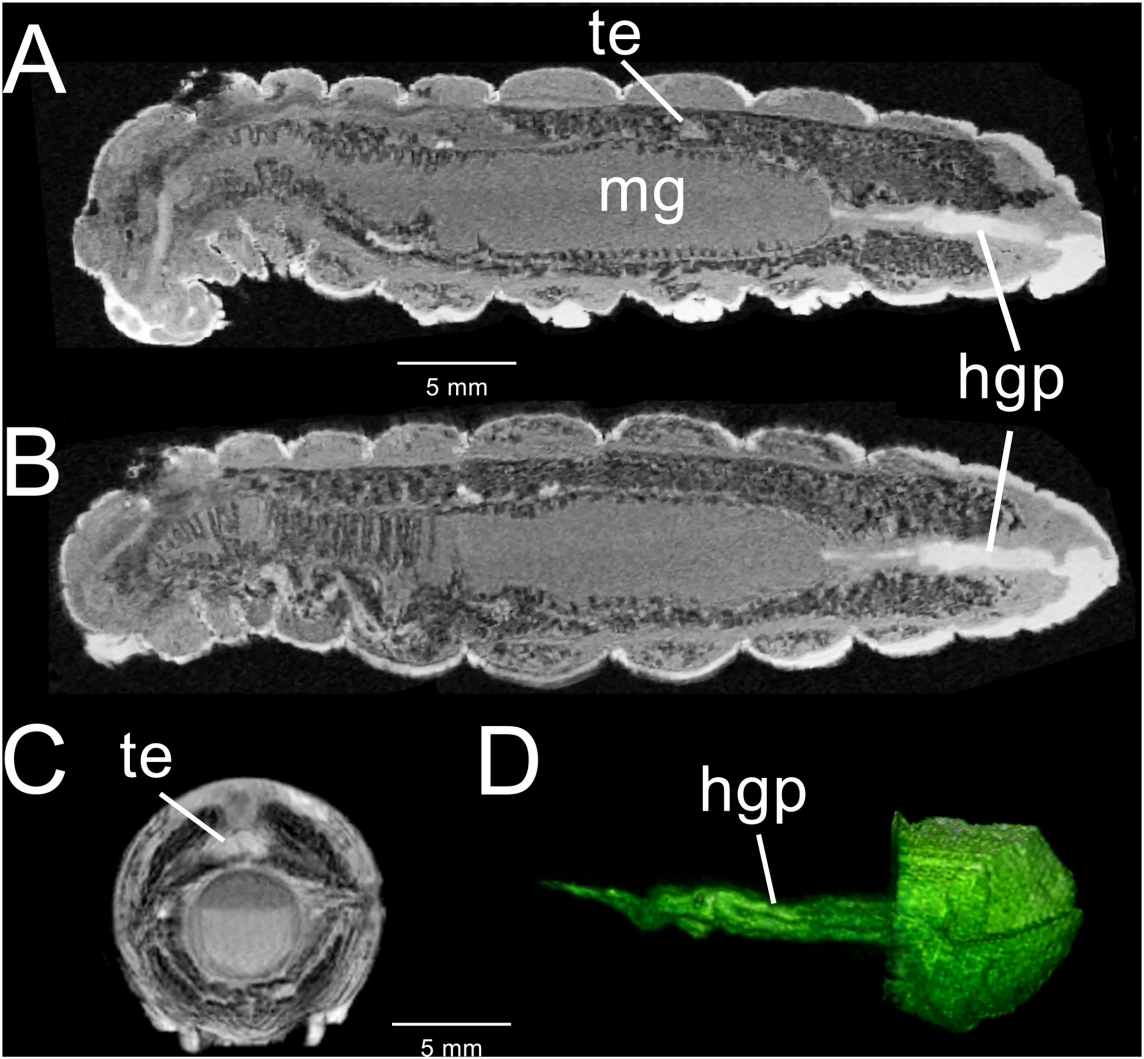
3D-GRE MR imaging data acquired from a male fifth instar *M*. *sexta* 12–16 hours before metamorphosis. (A) Mid-sagittal section displaying the testes (te), hindgut “plug” (hgp) and highly enlarged midgut. (B) Coronal section. (C) 2D-SE image showing a transverse section through the abdominal segment at the level of the testes. Layering in the midgut (mg) is clearly shown. Neither layer appears to contain gas bubbles. (D) Ventral 3D surface-rendered view of the hindgut “plug” and terminal abdominal segments.

Fig 3 illustrates the dramatic changes in the alimentary tract 12–16 hours before pupation, when the characteristic metathoracic bars appear [36]. The midgut, extending from the mesothoracic segment to abdominal segment 6, has become considerably swollen (Fig 3A and 3B). The anterior portion of the midgut is smaller in diameter than the posterior midgut. With fewer circular muscles, the corrugated outer surface of the midgut has changed, creating larger evaginations. Both foregut and hindgut continue to display regions that are isointense with the exoskeleton (Fig 3A and 3B). A 3D surface rendering of the hindgut “plug” is shown in Fig 3D. The surface has longitudinal grooves that parallel the hindgut axis and may represent shed hindgut cuticle. The transverse section of the midgut in Fig 3C indicates that the gut contents separate into two distinct phases. Upon dissection of age-matched larvae, the gut contents appear to be homogenous suspensions of amorphous particulates. While each layer in the images appear homogeneous and lack the gas bubbles seen in the feeding stage, the separation into two layers appears to be due to immobilization during image acquisition.

### Pupal stage: Day 2–3

**Fig 4:**
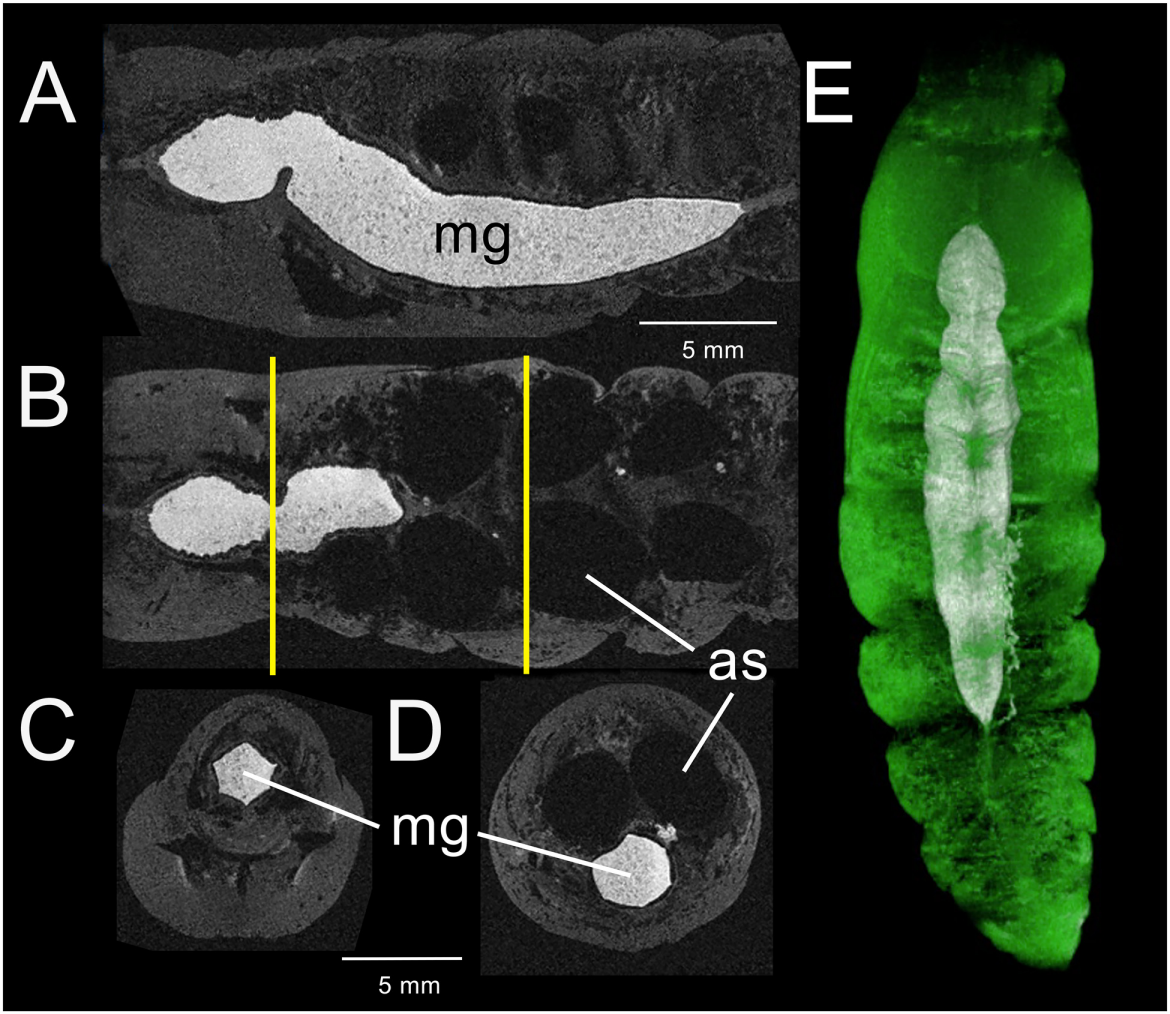
3D-GRE MR imaging data acquired from a *M*. *sexta* pupa 2–3 days after metamorphosis. (A) Sagittal view of thorax and abdomen displaying the enlarged midgut (mg) and newly formed air sacs (as). The contents of the midgut appear heterogeneous and lack gas bubbles. (B) Coronal view of the same segments, with air sacs prominent. (C) Transverse section through the thoracic-abdominal junction. Left line in (B) indicates level of section. (D) Transverse section through abdominal segment 5 displaying the enlarged air sacs that overlie the midgut. Right line in (B) indicates level of section. (E) Dorsal 3D surface-rendering showing the CA-enhanced midgut together with remnants of the right silk gland. Green regions overlying the midgut represent heart chambers.

Rudiments of the proboscis, cibarium and sucking pump [37] appear in gross dissections of the head region (not shown). The proboscis appears as a thin line of cells attached to trachea that extend into the external proboscis structure. The anterior midgut remains embedded in the thoracic region (Fig 4A, 4B and 4E), where flight muscles will develop. A constriction develops ventrally that begins to pinch off the anterior region from the rest of the midgut. The abdominal portion of the midgut migrates from its central core position to a more ventral aspect. At the same time, the diameter of the tract begins to narrow (Fig 4C and 4D). Segmental air sacs are now apparent in the dorsal abdomen (Fig 4A, 4B and 4D). The hindgut region does not appear to have changed significantly.

### Pupal stage: Day 5–7

**Fig 5:**
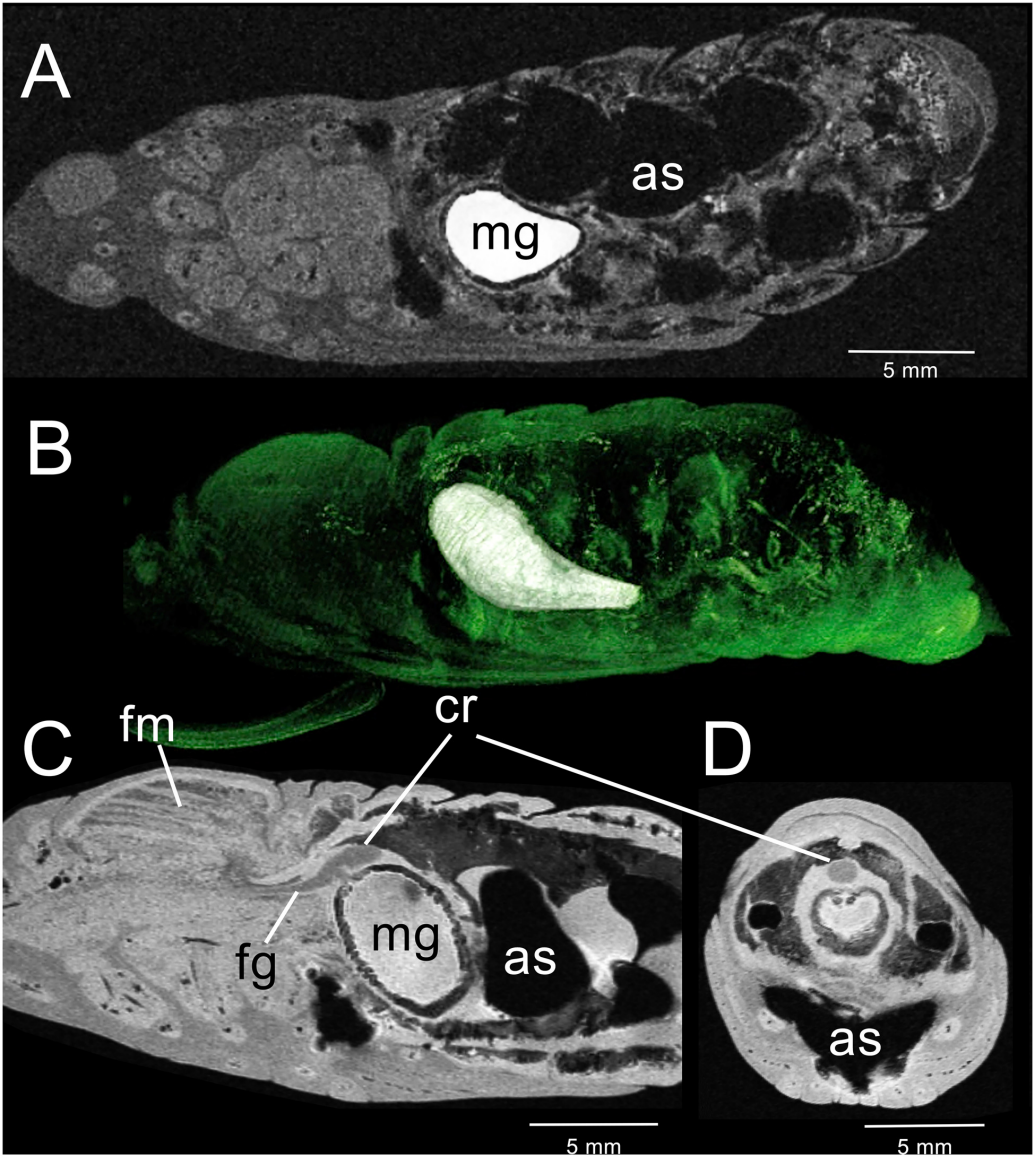
MR imaging data acquired from a *M*. *sexta* pupa 5–7 days after metamorphosis. (A) 3D-GRE coronal section displaying the enlarged air sacs (as). (B) Sagittal volume rendering view displaying the position of the midgut (mg, containing contrast agent) and developing hindgut (not containing contrast agent). (C) 3D-FSE sagittal section displaying the developing thoracic flight muscles (fm), midgut and crop (cr). The newly formed crop expands from the foregut (fg). (D) Transverse section through the crop and anterior midgut.

The shape of the midgut now resembles that of the adult. Continued expansion of the abdominal air sacs may play a role in relocating the midgut to its adult position (Fig 5A and 5C). The midgut has retreated to abdominal segments 2–4 and forms a bulbous structure, broad in the anterior region and tapering as it progresses toward the hindgut (Fig 5B). At day 5, gross dissection reveals that the pinched-off section of the anterior midgut has collapsed upon the remaining anterior midgut (not shown). By day 7, the pinched-off section appears to be fully incorporated into the anterior portion of the midgut. Concurrent with these changes is the elongation of the foregut as the midgut retreats into the abdomen (Fig 5C).

The hindgut begins to expand in length and coil (Fig 5B). Although the insect in Fig 5 was fed high doses of CA, much of it was excreted at gut purging during the fifth stadium. Since residual CA is present in the midgut but absent from the hindgut, we presume that the hindgut is physiologically isolated from the midgut. At this time, the rectal sac appears as a small outgrowth of the hindgut (not shown). The structure forms a T-shaped junction that projects anteriorly as well as posteriorly.

Fig 5C and 5D shows the developing crop at the posterior terminus of the foregut. Gross dissection at day 5 indicates that the crop originates from the dorsal region of a cone-shaped structure just anterior to the foregut-midgut junction. By day 7, the rapidly expanding crop is clearly visible by MR (Fig 5C and 5D). It continues to expand posteriorly along the dorsal contours of the midgut during the next 10 days. Elements of the adult proboscis, cibarium and sucking pump become evident between days 5 and 9.

### Pupal stage: Day 9–12

By day 9, most of the structures of the alimentary tract appear adult in nature (not shown). Gross dissection indicates the proboscis, cibarium and sucking pump are partially formed. The proboscis has expanded to its full length, folding back upon itself and entering the ventral cavity between the developing legs and antennae. The foregut is invested with a translucent structure assumed to be the pharate adult foregut. The crop has developed outward as a nearly transparent, highly folded sac that follows the dorsal contour of the midgut. More circular muscles in the midgut have broken down, reducing its corrugated appearance. The anterior portion of the hindgut continues to coil; the central portion of the rectal sac begins to fill, leaving a nipple-like structure facing anteriorly.

### Pupal stage: Day 16–17

**Fig 6:**
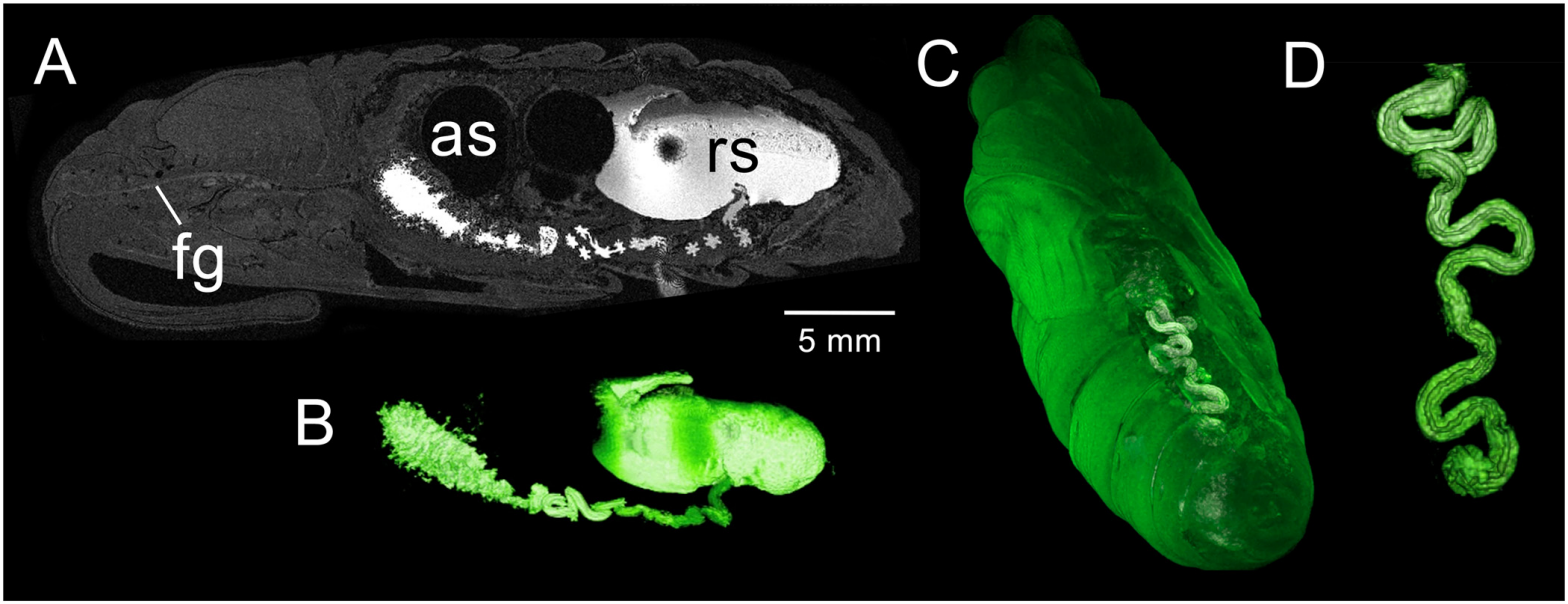
3D-GRE MR imaging data acquired from a *M*. *sexta* pupa 16–17 days after metamorphosis. (A) Sagittal section displaying the deflated midgut and enlarged rectal sac (rs). Abdominal air sacs (as) deform the anterior portion of the rectal sac. Midgut contents have been released into the hindgut. (B) 3D surface-rendering of the midgut, anterior hindgut and rectal sac. (C) Ventral 3D surface-rendering showing the position of the anterior hindgut. (D) 3D surface-rendering of the coiled anterior hindgut.

At day 16–17 CA, found previously only in the midgut, is present in the anterior hindgut and rectal sac (Fig 6A and 6B); presumably the midgut and hindgut are now physiologically connected. The anterior portion of the hindgut is highly coiled and the rectal sac continues to expand (Fig 6B–6D). In cross-section the anterior hindgut is stellate; the anatomy is probably analogous to that observed in the larval hindgut (compare Fig 6A to Fig 1F). The continued expansion of the rectal sac appears to be deformed by an air sac (Fig 6A). Fig 6C is a 3D surface-rendering of the pupa, showing the location of the anterior hindgut.

### Pupal stage: Day 22–23

**Fig 7:**
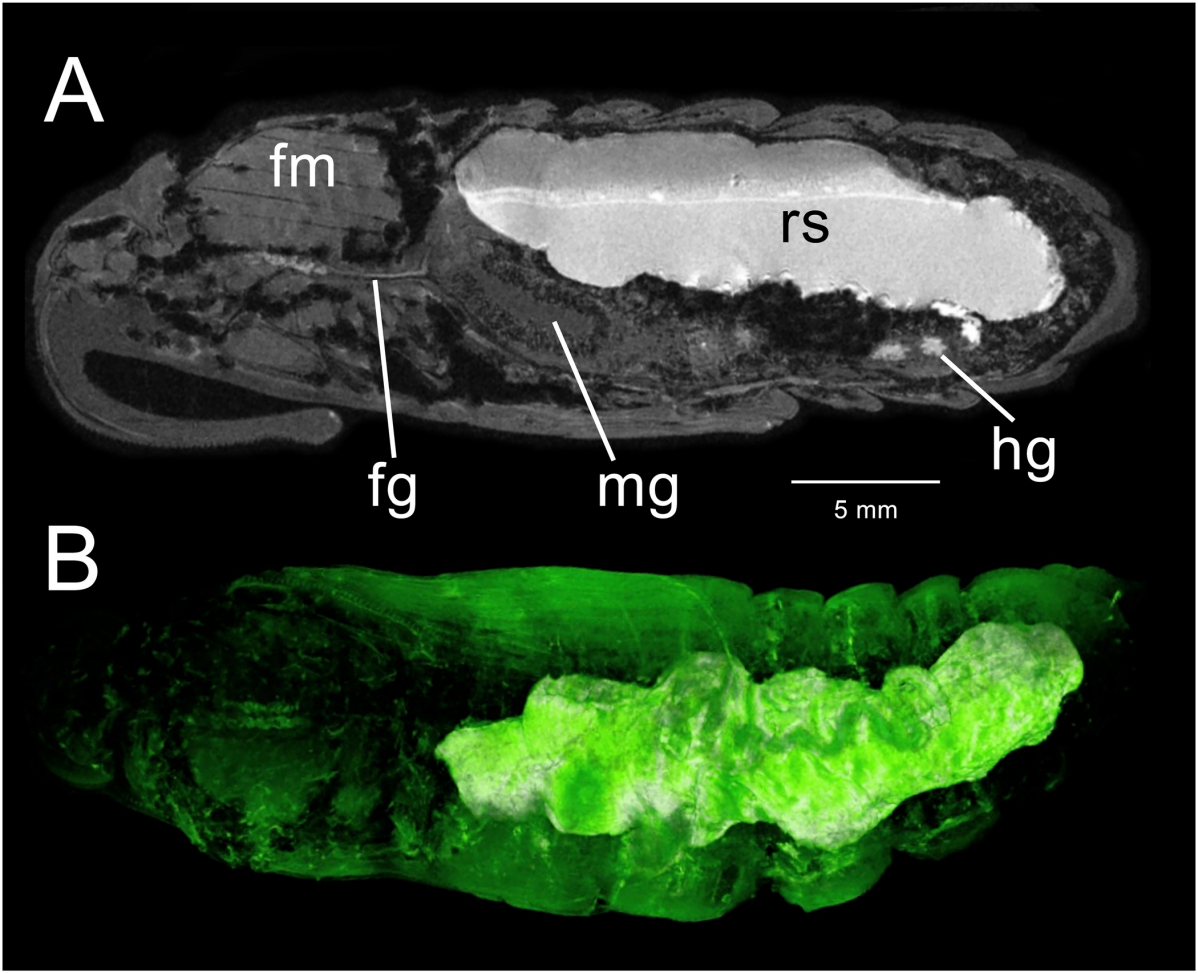
3D-GRE MR imaging data acquired from a *M*. *sexta* pupa 22–23 days after metamorphosis, approximately 12 hours before adult emergence. (A) Mid-sagittal view. The midgut (mg) is emptied of residual CA and the rectal sac (rs) spans the length of the abdomen. Flight muscles (fm) appear to be fully formed. Dark regions begin to appear in the thorax. (B) Ventral 3D surface-rendering showing the rectal sac and the outline of the anterior hindgut (hg). The midgut is not visible in this image.

The sucking pump is now a prominent structure in the head (Fig 7A). Gross dissection reveals the posterior portion of the foregut and semi-inflated crop are filled with a dark-brown liquid and air sacs around the midgut are inflated. Diffuse dark regions in the anterior dorsal thorax are indicative of significant air presence in the tracheae (Fig 7A). Residual contents of the midgut and anterior hindgut have moved into the rectal sac (Fig 7A and 7B). While the absence of CA in the midgut indicates that the midgut has emptied its contents into the hindgut, the midgut does not appear completely deflated. Most striking is the growth of the rectal sac, which now occupies much of the dorsal abdomen. When viewed from the ventral aspect, the rectal sac is in apposition to the highly coiled anterior hindgut. The rectal sac continues to expand in volume until pupal-adult eclosion. Images of the rectal sac reveal its contents to be separated into two layers. Upon dissection, it was found that the denser (upper) layer is composed of a fine, cream colored precipitate while the less dense layer (lower) is a dense, dark clear fluid. The pupa was imaged lying on its ventral side but is inverted in Fig 7 to be consistent with other images.

### Adult stage

**Fig 8:**
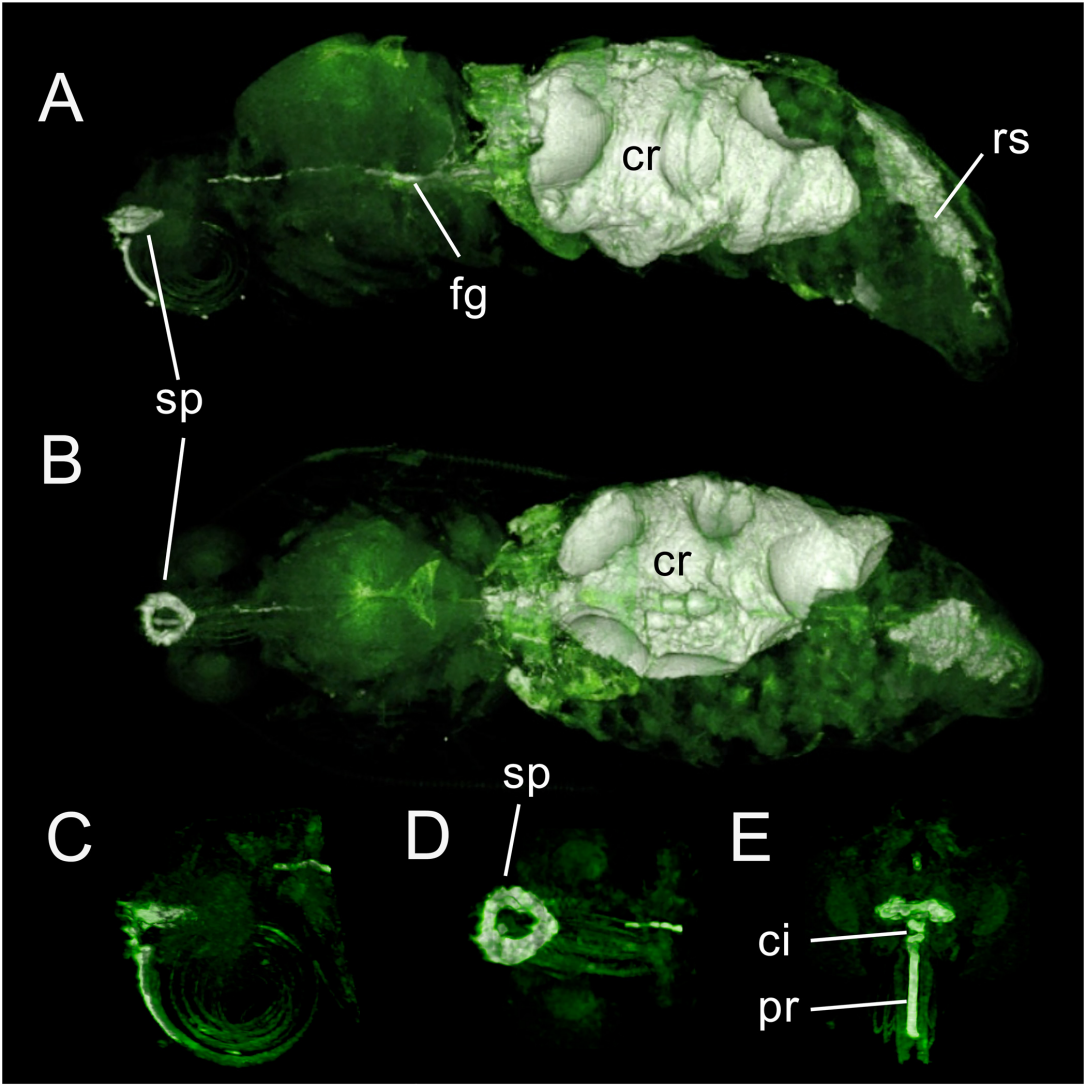
3D-GRE MR imaging data acquired from a *M*. *sexta* adult female. (A) Dorsal 3D surface-rendered view. Air sacs and ovarioles induce irregularities in the crop (cr) surface. Proboscis (pr), cibarium (ci), sucking pump (sp) and foregut (fg) are visible; the midgut is hidden in this view. The rectal sac (rs) is now deflated. (B) Coronal view showing the sucking pump, foregut and crop. The anterior portion of the midgut lies immediately anterior to the crop. Darkened circular structures in the abdomen are eggs. (C) Mid-sagittal view of the proboscis, cibarium, sucking pump and foregut. The cibarium lies just ventral to the sucking pump. (D) Dorsal view of the sucking pump. (E) Frontal view of the proboscis, cibarium and sucking pump.

Fig 8 illustrates an adult female that has been fed CA to enhance visualization of the alimentary tract. The midgut lies pressed ventrally and anteriorly by air sacs (not shown) and lies in the same relative position as the late pupal midgut. The midgut is nearly hidden by the inflated crop (Fig 8A and 8B); however, the anterior most portion is visible. The irregular bulging of the crop is due to segmental air sacs lying just beneath. The rectal sac, the bright abdominal structure just below the dorsal vessel (Fig 8A and 8B), has collapsed upon expulsion of the meconium. The reduction creates space in which the eggs can mature. Fig 8C represents a lateral view of the proboscis and head, indicating the position of the cibarium and sucking pump. Fig 8D is a dorsal view outlining the sucking pump. The sucking pump is partially deflated, giving rise to the dark area in the center that represents the buccal compressor. A frontal view shows the alimentary tract, the cibarium and the partially compressed sucking pump (Fig 8E).

### Development of auxiliary structures: Silk and mandibular glands

**Fig 9:**
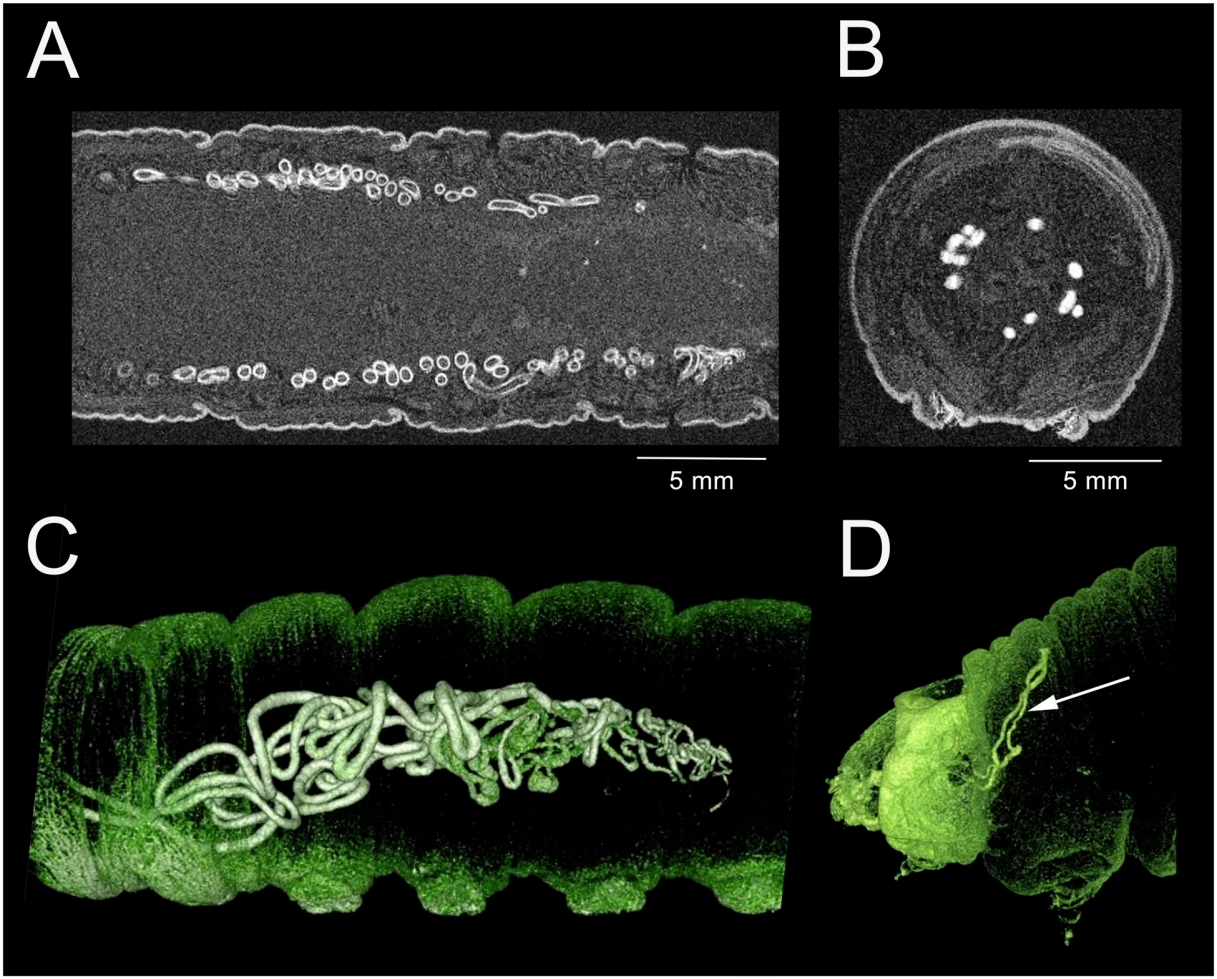
3D-GRE MR imaging data illustrating the silk and mandibular glands from 5th instar *M*. *sexta*. The insects shown were not fed contrast agent. (A) Coronal slice of a 108 hour larva, showing the silk glands. The ring-like appearance of the glands suggests the intracellular presence of a paramagnetic moiety. (B) Transverse section through a 180 hour larva, showing filled silk glands. (C) Lateral 3D surface-rendering of abdominal segments of a 204 hour larva, showing the structure and position of the silk glands. (D) Lateral 3D surface-rendering of the head and thorax of a 130 hour larva, showing the hyperintense mandibular glands.

The silk glands [38], also termed labial glands [39], are barely visible by MR during the feeding stages; however, following cessation of feeding the glands become hyperintense (Fig 9A–9C). Electron paramagnetic resonance analysis (EPR; data not shown) indicates that the hyperintensity of the glands is due to the presence of paramagnetic manganese species.

Maximum intensity is reached after the insect enters the wandering stage; afterwards, intensity progressively declines. Shrinkage of the glands begins distally and progresses anteriorly, suggesting that material is being secreted (Fig 9C). The mandibular glands are not MR-visible during the feeding stages; however, by 130 hours they too become hyperintense without the aid of CA (Fig 9D).

### Overview of alimentary tract development

**Fig 10:**
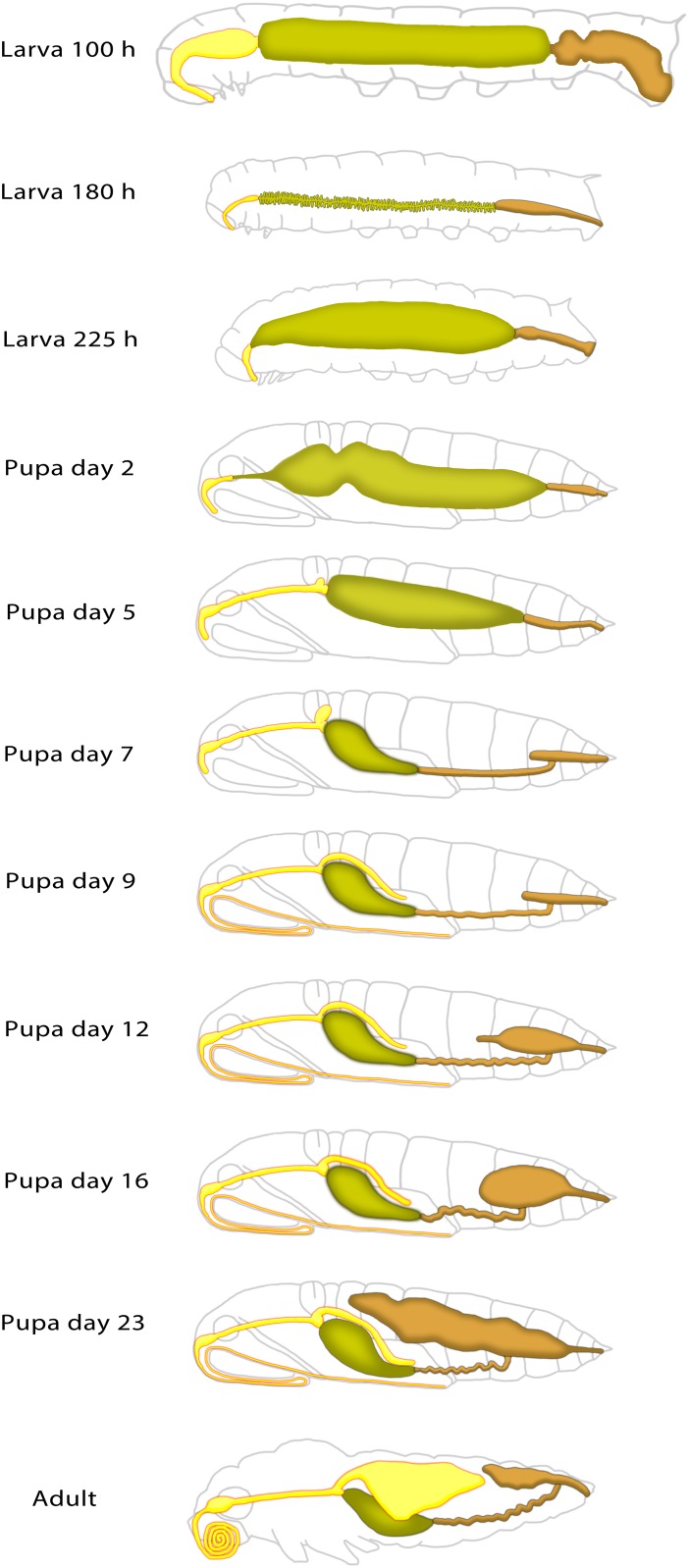
Diagrammatic representation of the developmental changes of the alimentary tract in *Manduca sexta* during the transition from its foliage feeding larval form through to nectar feeding moth.

Using MR imaging data combined with structural information obtained visually via gross dissection, the development of the alimentary tract during larval, pupal and adult stages may be shown diagrammatically (Fig 10). The diagram reveals the dramatic morphological changes undergone by *Manduca sexta* as it transitions from it larval form feeding on foliage to becoming a nectar feeding moth.

## Discussion

MRI offers a powerful tool to better understand the macroanatomical changes that occur during metamorphosis of the insect alimentary tract. Being non-invasive, MRI enables the acquisition of 3D data while avoiding organ disruption or distortion by chemical fixation or dissection, thereby preserving the macroanatomical context. With appropriate contrast agents and post-acquisition software, acquired images can provide dynamic visualizations of physiological processes such as hemolymph flow [26]. The developing alimentary tract of several Lepidopteran species has been extensively examined histologically [9, 11]. Here, MRI analyses, supported by gross dissection, focus on the alimentary tract of *Manduca sexta* at key developmental points from late larval to adult stage. Fig 10 pictorially summarizes the dramatic metamorphic changes observed.

### Foregut development

Restructuring the foregut to accommodate the radical change in diet from plant material to nectar begins soon after the cessation of larval feeding and is essentially completed by day 9 of the pupal stage. During pharate pupal development, the foregut shrinks and the lumen walls become crenulated [10]. The foregut lumen appears isointense with the exoskeleton, suggesting that the foregut lumen and the exoskeleton are similar in composition [40]. Dissection of the foregut as early as 132 hours after ecdysis to the fifth stadium revealed a transparent membrane that may be the shed cuticular intima. Thus, foregut ecdysis precedes molting of the exoskeleton by some 100 hours.

The MR data in conjunction with the dissection results clearly suggest that the foregut has already ecdysed by the time ecdysteroids reach their peak levels at approximately 180 hours of the fifth stadium (42). Based on this new evidence, foregut ecdysis must be initiated by the “commitment burst” [41] of ecdysteroids that occurs at approximately 108 hours (42). Thus, the commitment burst of ecdysteroids responsible for ending larval feeding and inducing burrowing behavior [42] may also be responsible for initiating ecdysis of the foregut and hindgut.

At day 5 of the pupal stage, the dorsal region immediately adjacent to the foregut-midgut junction forms a cone-shaped projection that will grow in size and become the crop. Previous investigators, noting cytological differences between this region and other parts of the foregut and midgut, have termed the region the anterior imaginal ring [11], [43, 44]6, [45]. Development of the crop from the anterior imaginal ring correlates with the dramatic rise in ecdysone titers between days 4 and 6 of the pupal stage [46, 47]. Warren and Gilbert (48) suggest that at times ecdysone may act as a hormone itself rather than a precursor to 20-hydroxyecdysone; this correlation between crop development and rise in ecdysone titers may be an example.

### Changes in the midgut

The midgut of the feeding larva is invested with two layers of muscles: circular muscles, which are immediately adjacent to the gut wall, and a series of 6 overlying bands of longitudinal muscles that run the length of the midgut [38, 48]. MRI of transverse sections of the midgut displayed two forms, circular and hexagonal. We surmise that midgut lying directly under a circular muscle will maintain the hexagonal form in a transverse section while midgut lying between the circular muscles will maintain a circular form even during peristalsis. Eaton [38], while not addressing these structural differences, did note that midgut wall between the bands of circular muscles folds outward, effectively increasing surface area. The result of this folding leads to the corrugated appearance of the midgut when viewed from the hemolymph side [48].

MRI analyses revealed that the anterior portion of the midgut in abdominal segments 1–3 is thrown into 6 out-pocketings by the longitudinal muscles. Previous studies on the larval midgut of *M*. *sexta* did not report these out-pocketings [38, 48, 49], as in dissection they are shrouded by a layer of closely adhering fat body. These out-pocketings resemble the anterior midgut structures observed in another moth, *Heliothis virescens* [50]. Judy and Gilbert (9), in their study on *Hyalophora cecropia*, did not observe these out-pocketings but did report the presence of multiple caeca-like protuberances attached to the anterior midgut. While these structures have not been observed in Lepidopterans generally (5), in some insect orders similar structures in the foregut or anterior midgut house bacteria. Whether the out-pocketings in *M*. *sexta* play such a role will need investigation.

In addition to displaying out-pocketings, MRI revealed that the anterior midgut appears more rugose than the rest of the midgut. Cioffi [48] noted cellular differences in the anterior midgut in her histologcal study on *M*. *sexta*. The midgut wall is composed of epithelial cells that are attached to a thin basement lamina [48]. The degree of epithelial folding gives rise to distinct regions. Cioffi [48] suggests that the regions may have different roles in uptake of ions and nutrients.

Metamorphic changes in the midgut volume of *M*. *sexta* are striking. After cessation of feeding, the midgut empties and collapses, then re-inflates during the larval-pupal transition. The collapse has been reported in several Lepidopterans, including *Bombyx mori* [51] and *Hyalophora cecropia* [10, 11]. Expansion of the midgut is not limited to the abdomen, as this study as well as that of Lowe et al. [52] demonstrates. The midgut during the expansion phase pushes into the thorax. Re-inflation of the midgut might be attributed to imbibed air or liquid. Given the appearance of material in the midgut, the MR data clearly show that inflation is not due to the swallowing of air. In their study on *M*. *sexta* lysozyme production, Russell and Dunn [53] observed an increase in the fluid volume of the midgut. Cornell and Pan [54] suggested that imbibed molting fluid contributes significantly to the liquid volume of the midgut in *M*. *sexta* pharate pupae. Fluid management is clearly an important element in reshaping the midgut and the metamorphic process as a whole.

The motive forces that restructure the midgut are both internal and external. The midgut cells change in shape from cuboidal to columnar, reducing the surface area of the epithelium [11], 52, [55]. Outside the digestive tract, the development of enlarged air sacs plays a significant role in repositioning the tract. Large air sacs are not present in larvae but appear in some species soon after the larval to pupal molt [30]. Kaneko [30], employing MRI on the butterfly *Pieris brassicae*, demonstrated that a large cavity appears ventrally that may push the midgut dorsally. A similar series of air sac movements has been reported in the butterfly *Vanessa cardui* [52]. Hallock [26] used fast-imaging with steady-state free precession MR techniques to demonstrate that the air sacs in *M*. *sexta* inflate and deflate relatively quickly. Hallock suggested this promotes movement of hemolymph but did not address the role of air sacs in positioning the midgut. The appearance of flight muscles at day 7 (Fig 5C), combined with hemolymph movement and air sac inflation/deflation, may provide the motive force to orient the midgut into its final position.

### Hindgut development

Shortly after cessation of larval feeding, a “plug” displaying deep longitudinal grooves appears within the hindgut lumen; these striations are similar to those observed on the surface of frass pellets. The “plug” has a MR signal isointense to that observed in the exoskeleton. Thus, we hypothesize that the “plug” represents shed cuticle. Judy and Gilbert [10] noted this structure in *H*. *cecropia* and termed it a pyloric plug. The hindgut as well as the foregut undergoes ecdysis earlier than the external exoskeleton, indicating these epidermal tissues are more sensitive to ecdysteroid titers than the external epidermis. The curious increase in juvenile hormone titers at this time [56] may slow ecdysis of cuticle not part of the alimentary tract.

Morphological and physiological changes in the pupal hindgut begin at day 5–7, when the hindgut starts to lengthen and coil. In their study on *H*. *cecropia*, Judy and Gilbert [10] observed that the elongation is initiated at the midgut-hindgut junction. Henson [44], in his study on *Vanessa urticae*, suggested that the tissue in this region is embryonically similar to that forming the crop. By day 16–17 the midgut, which was physiologically isolated from the hindgut, releases its contents. Judy and Gilbert [10] suggest that movement of midgut contents into the hindgut at this time is aided by peristaltic contractions of the midgut.

The most dramatic change in hindgut morphology begins approximately day 7 with the growth of the rectal sac (Fig 10). The MR data indicate that the rectal sac contributes greatly to the internal volume of the pharate adult; at eclosion, contents of the rectal sac constitute nearly 30% of the wet weight [57]. Interestingly, development of the rectal sac coincides with a large increase in hemolymph 20-hydroxyecdysone titer [46, 47]. Suzuki et al. [58, 59] demonstrated that expansion of the rectal sac in *Bombyx mori* requires the continuous presence of ecdysteroids.

### Changes in the silk and mandibular glands

The hyperintensity of both silk and mandibular glands in the absence of contrast agent suggests that these glands contain a high level of paramagnetic material. X-band EPR analysis of silk glands revealed the presence of paramagnetic manganese species (data not shown). Spectra typical of Mn-containing metalloproteins were obtained. The biological relevance of this finding is unclear and investigations into this phenomenon continue.

This MR study provides unique insights into the developmental changes in the alimentary tract of a single insect, *Manduca sexta*. The dynamic nature of the morphological changes of the alimentary tract during the insect’s transition from the 5^th^ instar larval stage through pupation and into its adult stage is described for the first time while relating those changes with endocrinological events. The use of MRI offers a powerful, non-invasive tool to better understand insect anatomy during development. This study, focusing on the alimentary tract, relied primarily on feeding to introduce CA; other organ systems could be studied by introducing tissue-specific or nonspecific CA into the hemocoel.
